# Dual blockade of EGFR and ERK1/2 phosphorylation potentiates growth inhibition of breast cancer cells

**DOI:** 10.1038/sj.bjc.6602051

**Published:** 2004-07-27

**Authors:** D C Lev, L S Kim, V Melnikova, M Ruiz, H N Ananthaswamy, J E Price

**Affiliations:** 1 Department of Cancer Biology, University of Texas MD Anderson Cancer Center, Houston, TX 77030, USA; 2 Department of Immunology, University of Texas MD Anderson Cancer Center, Houston, TX 77030, USA

**Keywords:** mitogen activated protein kinase, epidermal growth factor receptor, HER2, targeted therapy

## Abstract

One of the major targets for breast cancer therapy is the epidermal growth factor receptor (EGFR) and related receptors, which signal via different signal transduction pathways including the mitogen-activated protein kinase (MAPK) pathway. This study determined whether there is a correlation between EGFR/HER2 status and MAPK (ERK1/2) phosphorylation in breast cancer cells, and how this affects the response to an inhibitor of the receptors. Expression of EGFR, HER2 and phosphorylated ERK1/2 were measured by immunoblotting in a panel of breast cancer cell lines. Several lines expressed high levels of pERK1/2, with no obvious correlation with the level of EGFR/HER2. The EGFR tyrosine kinase inhibitor PKI166 inhibited growth and induced apoptosis in some cells with high levels of growth factor receptors (MDA-MB-468, SUM149, SKBR3), but was less effective in cells that also had high basal ERK1/2 activity (MDA-MB-231). The combination of an inhibitor of MAPK signalling (U0126) and PKI166 produced significantly more inhibition and apoptosis than either agent alone. This suggests that constitutive activation of the MAPK pathway may bypass inhibition of EGFR/HER2 tyrosine kinases, and lead to insensitivity to agents targeting the receptors. However, inhibiting both EGFR/HER2 and MAPK signalling can result in significant growth inhibition and apoptosis of EGFR-expressing breast cancer cells.

The high mortality rate from breast cancer metastasis has led to the intensive search for molecular alterations contributing to metastatic progression, with the aim of designing targeted therapies (Dillon *et al*, 1998; Hortobagyi, 2000). Many of these are now in clinical trials (Kerbel, 2000; Mendelsohn and Baselga, 2000; Posey *et al*, 2001). Epidermal growth factor receptor (EGFR) (also known as erbB1) and HER2 (or erbB2) are two widely studied molecules that are prototypic members of the erbB family of tyrosine kinase receptors (Olayioye *et al*, 2000). Other family members are erbB3 and erbB4. Amplification of the *EGFR* or *erbB2* genes, leading to protein overexpression, occurs frequently in several human cancers. In breast cancer, *EGFR* or *erbB2* are overexpressed between 20 and 50% of cases, and increased expression is associated with shortened disease-free and overall survival, pointing to involvement in growth regulation of the tumours (Slamon *et al*, 1987; 1990; Lacroix *et al*, 1989; Klijn *et al*, 1992). The EGFR is an *M*_*r*_ 170 000 plasma membrane glycoprotein that dimerises upon binding ligand (e.g. EGF or TGF-*α*), resulting in activation of intrinsic tyrosine kinase activity and tyrosine autophosphorylation. This triggers a cascade of biochemical and physiological responses that constitute the mitogenic signal transduction of the cells. Extensive preclinical studies have shown that these signalling cascades regulate multiple cellular processes such as proliferation, differentiation, survival and transformation. Similar details of HER2 function are less well known and no soluble ligand for this receptor has been identified. However, this receptor plays a pivotal role in EGF-mediated signalling, as it is the preferred and most potent heterodimerisation partner for EGFR (Graus-Porta *et al*, 1997).

Several kinase cascades have been implicated in signal transduction through the erbB receptors (Moghal and Sterenberg, 1999). One of these is the RAS-RAF pathway, leading to the activation of extracellular signal-regulated kinases (ERKs), one of the mitogen activated protein kinase (MAPK) cascades (Campbell *et al*, 1995). This pathway has been the subject of intense interest because of its role in the regulation of proliferation, differentiation and cell–matrix interactions. ERK1 and ERK2 are dually phosphorylated (pERK) on threonine and tyrosine residues by mitogen-activated protein kinase/extracellular signal-regulated kinase kinase (MEK). Activated ERK1/2 phosphorylate and activate a variety of substrates including transcription factors, protein kinases and phosphotyrosine protein phosphatases, leading to positive or negative regulation of signalling cascades (Campbell *et al*, 1995). The mitogenic effect of EGF on normal and malignant mammary epithelia is dependent, at least in part, on ERK activation (Klapper *et al*, 2000). Furthermore, mammary tumour epithelium may exhibit an elevation in basal ERK activity and sustained ERK activation when stimulated by EGF (Xing and Imagawa, 1998). The sustained ERK activation may reflect a difference in the regulation of EGFR activity in the tumour cells *vs* normal breast epithelium. Elevated ERK1/2 activity has been noted in a proportion of clinical breast cancers *vs* benign disease or cancer-associated normal epithelium (Sivaraman *et al*, 1997; Santen *et al*, 2002), and a relationship between elevated MAPK activity and shorter disease-free survival in primary breast cancer has been reported (Mueller *et al*, 2000; Gee *et al*, 2001; Adeyinka *et al*, 2002). Thus, hyperactivity of ERK1/2 may play a role in breast cancer progression. It has been suggested that this increased activity is related to the pathological hyperexpression of EGFR and/or HER2 (Von Lintig *et al*, 2000). If so, the inhibition of the EGFR/HER2 tyrosine kinase activity should lead to a decrease in the pERK activity (Albanell *et al*, 2001). However, if the activation of pERK1/2 is independent of EGFR/HER2 signalling then increased pERK activity can theoretically bypass or over-ride, at least partially, the inhibition of these growth factor receptors.

Several agents that target one or more members of the ErbB family of tyrosine kinase receptors are currently undergoing preclinical and clinical investigations (Mendelsohn, 2001; Slichenmyer and Fry, 2001). In the present study we have used PKI166 {4-(R)-phenethylamino-6-(hydroxy)phenyl-7H-pyrrolo[2,3-d]-pyrimidine}, an inhibitor of the pyrrolopyrimidine class that has been shown to inhibit the intracellular domain of the EGF-R kinases with an IC_50_ of 0.7 nM, with less activity against other tyrosine kinases (Traxler *et al*, 2001). The goals of the present study were to characterise the pattern of expression of activated ERK1/2 in established breast cancer cell lines and determine whether this correlates with EGFR or HER2 status. Further, to explore whether activation status of ERK1/2 can be used as a marker of EGFR kinase inhibition and antiproliferative effects of agents targeting the growth factor receptors. Our results indicate that high basal activity of ERKs give some breast cancer cells a mechanism to escape the inhibitory effects of EGFR/HER2 tyrosine kinase inhibition. However, a combination of agents to inhibit both EGFR/HER2 and pERK signalling resulted in significant growth inhibition and induction of apoptosis.

## MATERIALS AND METHODS

### Breast cancer cell lines

A panel of breast cancer cell lines was used in an initial screen of cells to study further, based on expression of EGFR, HER2 and ERK activity. The lines were obtained from the American Type Culture Collection (Rockville, MD, USA) (T47D, SKBR3, Hs578T, MCF-7, ZR-75, BT-20), or provided by the Goodwin Institute (Plantation, FL, USA) (GI101A), Dr Stephen Ethier (University of Michigan) (SUM149), or Dr Relda Cailleau (UT MD Anderson Cancer Center) (MDA-MB-231, MDA-MB-435, MDA-MB-468, MDA-MB-361). Cells were maintained in medium (either MEM, or DMEM-F12) with 5 or 10% foetal bovine serum and L-glutamine, in a humidified incubator at 37°C with 5%-CO_2_. Cells of five cell lines (MDA-MB-231, MDA-MB-435, MDA-MB-468, SUM149 and GI101A) were injected in the mammary fatpads of nude mice, as described previously (Price, 1996). The resulting tumours were collected for preparation of tissue lysates. The use of animals was approved by the Institutional Animal Care and Use Committee of the University of Texas MD Anderson Cancer Center.

### Antibodies and inhibitors

Anti-total ERK1/2 and phosphorylated-p44/42 MAPK (Thr202/Tyr204) antibodies, anti-EGFR and pEGFR antibodies, anti-HER2 and pHER2, anti-p27^kip1^, and the MEK1/2 inhibitor UO126 were purchased from Cell Signalling Technology, Inc., Beverly, MA, USA. A polyclonal antibody to *β*-actin was purchased from Sigma Chemical Co., St Louis, MO, USA). Novartis Pharmaceutical (through Dr IJ Fidler, UT MD Anderson Cancer Center) provided PKI166, and a working solution of the tyrosine kinase inhibitor (5 mM in DMSO) was prepared immediately before use.

### *In vitro* growth

Breast cancer cells were plated in 96-well culture plates at an initial density of 2 × 10^3^ cells per well, and allowed to attach for 24 h. The culture medium was changed and the cells were incubated for a further 72 h in the following: medium alone, or with DMSO (0.1% vol vol^−1^), or PKI166 (0.5 or 5.0 *μ*M), or UO126 (10 *μ*M), or a combination of UO126 and PKI166. Relative cell numbers were determined using MTT. The conversion of MTT to formazan in metabolically viable cells was monitored with an MR-5000 microtiter plate reader reading at 570 nm (Dynatech, Inc Chantilly, VA, USA). All assays were performed in triplicate, with a minimum of three independent experiments.

### Measurement of apoptosis

Apoptosis was assessed by measuring DNA fragmentation by propidium iodide staining and FACS analysis (Nicoletti *et al*, 1991). Cells were incubated with PKI166 (0.5 or 5.0 *μ*M), with U0126 (10 *μ*M), or a combination for 48 h. The cells were harvested, pelleted by centrifugation and resuspended in PBS with 50 *μ*g ml^−1^ propidium iodide, 0.1% Triton X-100 and 0.1% sodium citrate. Samples were stored overnight at 4°C and vortexed before analysis.

### Immunoblotting

Expression of EGFR, HER2, pERK1/2, total ERK1/2 and p27^kip1^ were detected by immunoblotting. Protein lysates were prepared from cultures of cells that were at 80% confluency. For detection of the effect of PKI166 and/or UO126 on phosphorylation of EGFR and ERK1/2, lysates were prepared from cultures of cells that were grown in serum-free medium for 24 h prior to addition of the inhibitors. After 1 h incubation with the inhibitors, the cells were stimulated with 50 ng ml^−1^ EGF for 15 min, for assays of EGFR activation. For protein analysis of tumour lysates, fresh tumour tissue was homogenised in lysis buffer containing 50 mM Tris-HCl, 150 mM NaCl, 2 mM EDTA and freshly added inhibitors (1 mM Na_3_VO_4_, 1 mM PMSF, 1 *μ*g ml^−1^ pepstatin A, 2 *μ*g ml^−1^ aprotinin and 0.5 *μ*g ml^−1^ leupeptin). After homogenisation, NP-40 was added (1% vol vol^−1^) and the lysate was mixed, cooled on ice for 30 min, and centrifuged at 10 000 **g** for 10 min Aliquots of 20 mg (tumour lysate) or 20 *μ*g (cell culture lysate) of protein were separated on 7.5% SDS–polyacrylamide gels and electrophoretically transferred onto nitrocellulose membranes. The membranes were hybridised with a polyclonal antibody to EGFR, HER2, pERK1/2 or total ERK1/2, then incubated with an HRP-conjugated anti-rabbit IgG, and antibodies detected with the Amersham ECL system (Amersham, Arlington Heights, IL, USA), following the manufacturer's recommended procedure. Antibodies were removed by incubating filters in stripping buffer (62.5 mM Tris-HCl, pH 6.7, 2% sodium dodecyl sulphate, 100 mM *β*-mercaptoethanol). The filters were then hybridised with another primary antibody, or with antibody to actin to demonstrate equal loading and transfer of proteins. Immunoreactive bands were quantified by densitometry using ImageQuant software (Molecular Dynamics, Sunnyvale, CA, USA).

## RESULTS

### Differences in activity of ERK1/2 in the breast cancer cell lines

**Figure 1:**
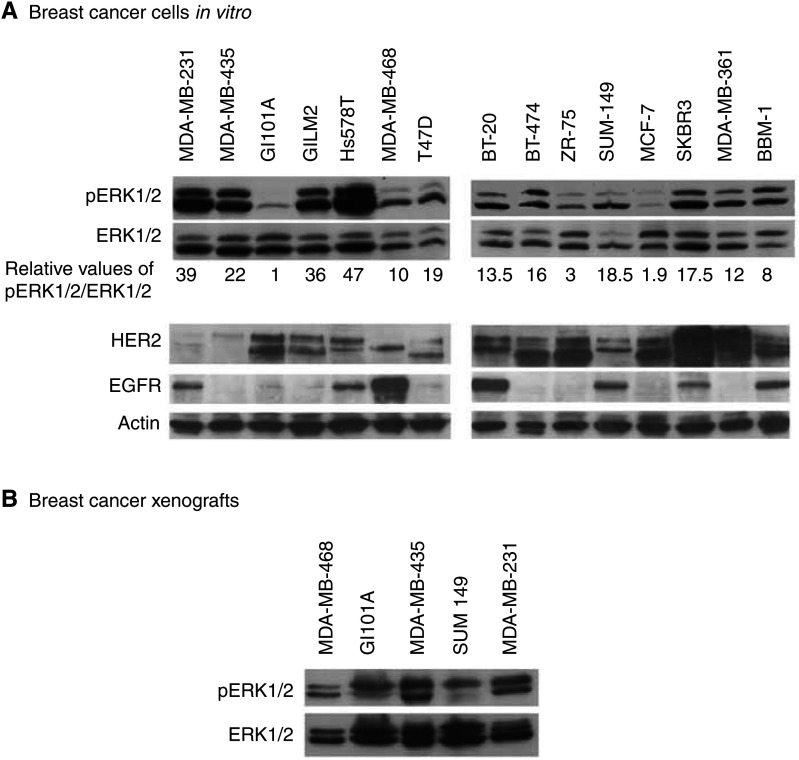
(**A**) Expression of EGFR, HER2, pERK1/2 and total ERK1/2 in lysates of breast cancer cell lines, determined by immunoblotting. Filters hybridised with antibody against pERK1/2 were re-probed with antibody against total ERK1/2. Densitometry of the immunoreactive bands generated the values shown under the ERK1/2 panel; these represent phosphorylation of ERK1/2 relative to total ERK1/2 and corrected for equal loading relative to actin (actin for the ERK1/2 hybridisations not shown). The pERK1/2/ERK1/2 values were then normalised to the value for GI101A cells, the line with the lowest value and assigned the value=1. The protein samples used for EGFR and HER2 and actin immunoblots were from different preparations, and are representative of the results from repeated experiments. (**B**) Immunoblot showing pERK1/2 and total ERK1/2 in lysates prepared from xenografts of human breast cancer cells.

Immunoblotting revealed differences in basal levels of ERK1/2 phosphorylation in different breast cancer cell lines, while the expression of ERK1/2 protein, normalised to actin expression, was relatively consistent (Figure 1A

). To test whether the ERK1/2 activity was only a tissue culture phenomenon, selected cell lines were injected into the mammary fatpads of nude mice, and protein lysates were prepared from the tumours. Taking into account the fact that lysates were of a mixture of tumour cells and surrounding stromal and infiltrating host cells, the immunoblotting of the tumour-derived proteins showed similar results to those obtained using lysates of cultured cells. MDA-MB-231 and MDA-MB-435 tumour lysates showed high levels of p-ERK1/2 in comparison to MDA-MB-468 and GI101A tumours (Figure 1B).

### Elevated ERK activity does not necessarily correlate with the status of EGFR and HER2 in breast cancer cells

Since ERK1/2 can be activated via EGFR and HER2 signalling, relative expression levels of these growth factor receptors were measured in the panel of cell lines, to test if there was a correlation between ERK activation and receptor expression levels. As expected from the heterogeneity seen in clinical specimens of breast cancer, there was variability in expression of EGFR, from high expression in MDA-MB-468 and minimal expression in MDA-MB-435 cells (Figure 1A). Comparing these results with the level of pERK1/2 indicated that there was no direct correlation between levels of these growth factor receptors and basal levels of ERK1/2 phosphorylation. Thus, while the MDA-MB-231 cell line with highly activated ERK1/2 expressed a relatively high level of EGFR, other combinations occur. High pERK1/2 levels were detected in MDA-MB-435 cells, which have very little EGFR, in contrast to the SUM149 cells with high EGFR expression and low ERK1/2 activity. Similarly, no correlation was found between the expression of HER2 receptor and the status of pERK (Figure 1A).

### PKI166 inhibition of breast cancer cell proliferation

Six cell lines with different levels of EGFR expression were selected for treatment with PKI166. Initial studies used a dose range of 0.1–5.0 *μ*M (data not shown), and the results of treating cells with 0.5 and 5.0 *μ*M are shown in Table 1

**Table 1:** Percent growth inhibition of cells of six breast cancer cell lines by the EGFR tyrosine kinase inhibitor PKI166 and the MEK inhibitor U0126

	**% Mean growth inhibition (s.d.)**				
	**U0**	**PKI (0.5 *μ*M)**	**PKI (0.5 *μ*M)+U0**	**PKI (5.0 *μ*M)**	**PKI (5.0 *μ*M)+U0**
MDA-MB-231	8.5 (1.1)	3.3 (0.5)	26.2 (3.5)^a^	33.7 (8.1)	61.5 (8.65)^b^
MDA-MB-468	24.3 (2.5)	21.1 (1.2)	54.2 (4.2)^a^	48.2 (6.8)	63.6 (4.2)^b^
SUM149	20.0 (8.9)	46.7 (13.8)	63.2 (5.2)^a^	70.8 (4.2)	80.7 (4.2)^b^
SKBR3	17.5 (1.3)	55.0 (15.5)	66.3 (4.6)	76.3 (5.3)	86.7 (6.4)^b^
MDA-MB-435	68.4 (8.2)	5.2 (2.1)	67.2 (6.3)^a^	32.2 (4.2)	74.0 (7.8)^b^
GI101A	12.0 (2.3)	13.7 (1.8)	10.0 (1.2)	18.5 (2.3)	23.5 (2.6)

Growth inhibition of cells cultured in the presence of PKI166 (0.5 or 5.0 *μ*M) or U0126 (10 *μ*M), or the combination for 72 h, calculated from MTT assays, and expressed as a percent growth inhibition compared with cells grown in the presence of DMSO (0.1%) as control. Values shown are mean values from triplicate wells, and are representative of repeated experiments.

a Inhibition by PKI (0.5 *μ*M) *vs* inhibition by PKI (0.5 *μ*M)+U0 (*P*<0.05).

b Inhibition by PKI (5.0 *μ*M) *vs* inhibition by PKI (5.0 *μ*M)+U0 (*P*<0.05), analysed using Student's *t*-tests.

. Growth inhibition was determined from the results of MTT assays, comparing PKI166 treated cells with cells exposed to medium with 0.1% DMSO. Treatment with 0.5 *μ*M PKI166, a concentration less than plasma and tumour concentrations achieved in preclinical models from oral administration of the drug, and the higher dose of 5.0 *μ*M, produced different levels of growth inhibition in different cell lines. As expected, cells expressing low levels of EGFR and HER2, GI101A, MDA-MB-435 showed least growth inhibition. However, not all of the high EGFR-expressing lines were sensitive to PKI166. The lower dose produced 46 and 21% growth inhibition of SUM149 and MDA-MB-468 cells, respectively, but had little effect (3.3% inhibition) on the growth of MDA-MB-231 cells. The SKBR3 cells, expressing EGFR and also high levels of HER2, were most sensitive, showing 55% growth inhibition with 0.5 *μ*M and 76% inhibition with 5.0 *μ*M PKI166.

### PKI166 inhibits phosphorylation of EGFR and HER2 in breast cancer cells

**Figure 2:**
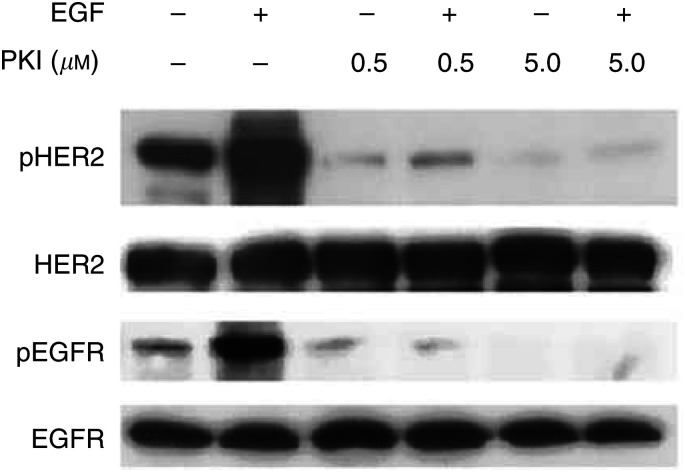
PKI166 inhibits phosphorylation of HER2 and EGFR in SKBR3 cells. The cells were serum-starved for 24 h before incubation with PKI166 (0.5 or 5.0 *μ*M) for 1 h, then stimulation with EGF (50 ng ml^−1^) for 15 min before preparation of protein lysates. Phosphorylation of the growth factor receptors was detected with antibodies recognising the activated forms of HER2 and EGFR. The same filters were then reprobed with antibodies recognising the ‘total’ receptors.

To demonstrate inhibition of EGFR and HER2 phosphorylation by the concentrations of PKI166 used for the growth inhibition assays, cell lysates were prepared from MDA-MB-231, MDA-MB-468, SUM149 and SKBR3 cells after treatment with PKI166 and stimulation with EGF, and phosphorylation of the receptors assessed. PKI166 inhibited ligand-induced EGFR phosphorylation in a dose dependent manner in these four cell lines, and also phosphorylation of HER2 in SKBR3 cells, in the absence or presence of 50 ng ml^−1^ EGF (Figure 2

) (data for other cell lines not shown).

### Constitutive ERK1/2 phosphorylation as a potential escape mechanism from inhibition by PKI166

Inhibition of growth by PKI166 was most effective in cells with high levels of EGFR and nonactivated ERK1/2 (SUM149, MDA-MB-468) when compared with cells with high EGFR and high basal level of phosphorylated ERK1/2 (MDA-MB-231). To test whether the basal ERK1/2 activity was providing an escape mechanism from inhibition by PKI166, cells were treated with a combination of PKI166 and UO126, an inhibitor of MEK (Table 1). GI101A cells, with low EGFR and nonactivated ERK1/2, showed modest growth inhibition when treated with an individual inhibitor and no significant difference with the combination of the two. MDA-MB-435 cells were significantly inhibited by U0126 alone, and the addition of PKI166 made no difference. The combination of agents significantly increased the antiproliferative action of PKI166 at the 0.5 and 5.0 *μ*M doses in cells expressing higher levels of EGFR or HER2 (SUM149, MDA-MB-468, SKBR3), including MDA-MB-231 cells. Treating the MDA-MB-231 cells with U0126 alone produced 8.5% inhibition, which was not significantly different from control values. The addition of U0126 to 0.5 or 5.0 *μ*M PKI166 significantly increased the growth inhibition produced by the receptor tyrosine kinase inhibitor alone (Table 1).

**Figure 3:**
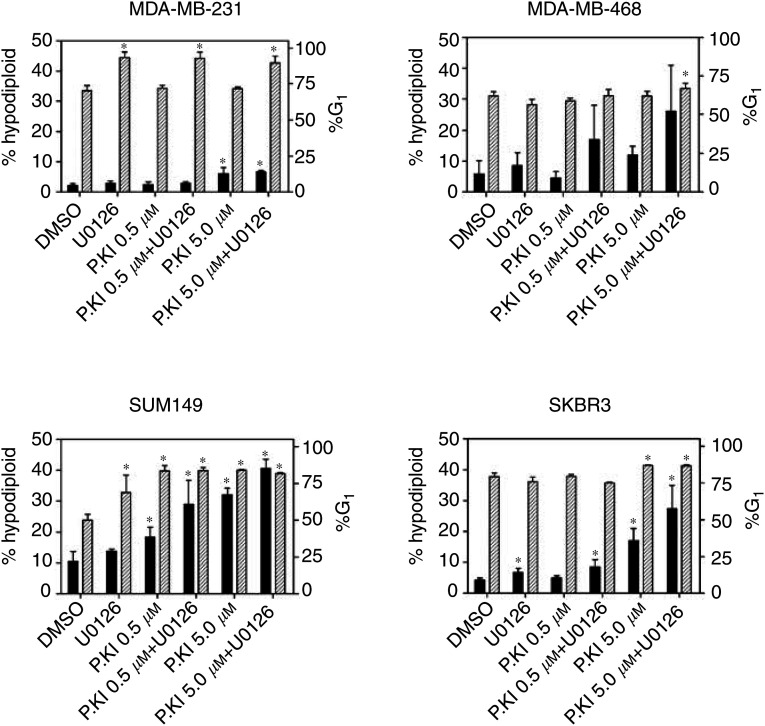
PKI166 and U0126 induce apoptosis and increase proportions of cells in G_1_. The percentages of cells in sub-G_1_/hypodiploid (solid bars) and in G_1_ (cross-hatched bars) following 48 h treatment with PKI166 (0.5 or 5.0 *μ*M) or U0126 (10 *μ*M), and the combination were determined by FACS analysis of propidium iodide stained cells. Values shown are the mean and SD from three independent experiments, and ^*^ indicates a significant difference from the control values, *P*<0.05, Student's *t*-test.

**Figure 4:**
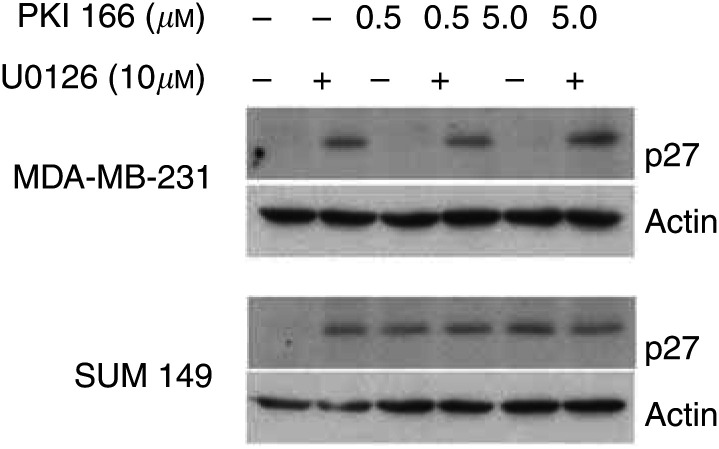
Induction of p27^KIP1^ by PKI166 and U0126, detected by immunoblotting lysates of cells from the same experiment described in Figure 3. Either inhibitor induced expression of the cyclin-dependent kinase inhibitor in SUM149 cells, while only U0126 produced detectable protein in MDA-MB-231 cells.

Apoptosis induced by PKI166 and U0126 was assessed by measuring DNA fragmentation by propidium iodide staining and FACS analysis, and determining the proportions of hypodiploid cells. This showed that PKI166 alone or in combination with U0126 induced apoptosis in the EGFR or HER2 expressing cell lines MDA-MB-231, MDA-MB-468, SKBR3 and SUM149 cells (Figure 3

), although the proportions of hypodiploid cells varied between the different lines. Similar to the MTT results in Table 1, SKBR3 and SUM 149 cells were most sensitive to treatment with the inhibitors, while the proportions of hypodiploid MDA-MB-231 cells were lower. Treatment with U0126 alone significantly increased the numbers of MDA-MB-231 in the G_1_ phase of the cell cycle (89–93% compared with 70–72% of control or PKI 166 treated cells, *P*<0.001). The proportion of SUM149 cells in G_1_ was significantly increased by treatment with either inhibitor alone and the combination, while apoptosis was significantly increased in cells exposed to PKI166, with or without U0126. Induction of the cyclin-dependent kinase inhibitor p27^KIP1^ generally corresponded with increases in the proportion of cells in G_1_, as shown for MDA-MB-231 and SUM149 (Figure 4

).

### Differential effect of PKI166 on ERK1/2 phosphorylation

**Figure 5:**
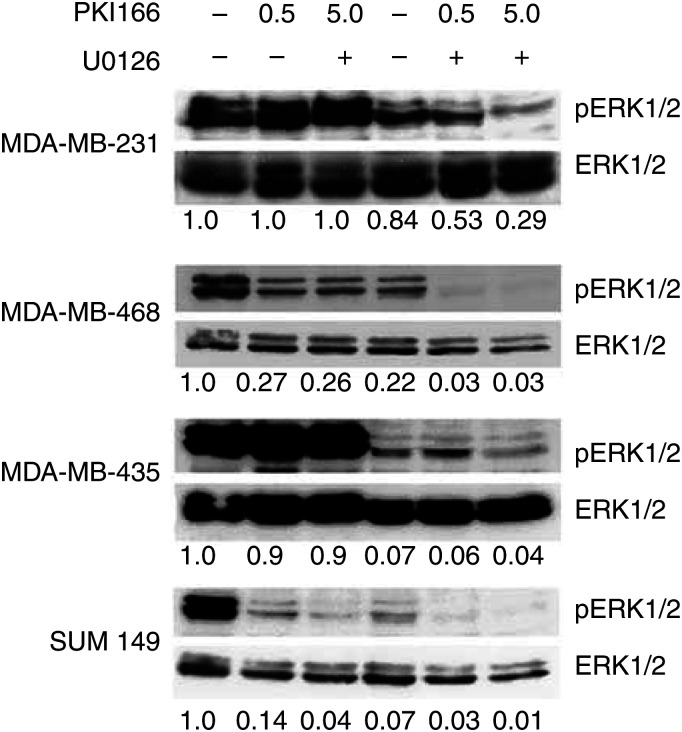
Immunoblots showing pERK1/2 and total ERK1/2 in lysates of cells serum-starved for 24 h, then treated with PKI166 (0.5 or 5.0 *μ*M) or UO126 (10 *μ*M) as indicated, for 1 h before preparation of the lysates. Values of the relative expression of pERK1/2 expressed as a ratio of total ERK1/2 expression, and corrected for equal loading relative to actin (not shown) indicated below the immunoblots were determined by densitometry, and expressed as proportion of the control values (cells treated with DMSO).

To evaluate whether the antiproliferative effects of EGFR inhibition involve ERK1/2 activation, the status of pERK1/2 was determined in cells exposed to the same concentrations of PKI166 used for the growth inhibition assays, in the presence and absence of U0126 (10 *μ*M) (Figure 5

). U1026 alone inhibited ERK1/2 phosphorylation in MDA-MB-435 cells, with PKI 166 having no effect, as expected from minimal expression of EGFR in these cells. PKI166 inhibited ERK1/2 phosphorylation in SUM149 cells, as did U0126 alone, and further inhibition by the combination of drugs was barely discernible. Treatment of MDA-MB-468 with either drug resulted in similar inhibition of ERK1/2 phosphorylation, with almost complete elimination of phosphorylated proteins by the combination. PKI166 alone minimally altered the ERK1/2 status in the MDA-MB-231 cells, and U0126 produced some inhibition, while the combination resulted in a substantial reduction, reflecting the effect on cell proliferation and apoptosis. For SUM149 and MDA-MB-468 cells the combination of the inhibitors almost completely eliminated ERK1/2 phosphorylation after 1 h incubation, although growth inhibition over 72 h was 54–63% with 0.5 *μ*M PKI166 plus 10 *μ*M U0126, and 63–81% with 5.0 *μ*M PKI166 plus 10 *μ*M U0126 (Table 1). Recovery of ERK1/2 phosphorylation in the U0126-treated cells over the period of the growth inhibition assays was not investigated, but the data may also suggest that other signal pathways were contributing to the growth and survival of the cells. The effects of the inhibitors were not related to downregulation of total ERK1/2 proteins, as the levels did not decrease with treatment (Figure 5).

## DISCUSSION

Due to growing disappointment with current therapies for breast cancer, which have not lead to significant alleviation of metastatic disease, more attention has been focused on developing novel targeted therapies (Bange *et al*, 2001). Major targets are members of the erbB family of growth factor receptors, principally EGFR and HER2, which are known to play important roles in breast cancer pathogenesis (Lacroix *et al*, 1989; Slamon *et al*, 1990; Klijn *et al*, 1992). As the targeted agents move into clinical trials it will be important to have a clear understanding of the EGFR-dependent pathways and their patterns of expression and activation. The present study focuses on the relationship between EGFR and the MAPK pathway. This choice was not arbitrary, as this pathway is one of the major downstream signalling pathways from EGFR. Activated ERK1/2 control many processes that are central to malignant progression, including cell growth, apoptosis and migration (Pages *et al*, 1993; Campbell *et al*, 1995; Hoshino *et al*, 1999; Schramek, 2002).

Disrupting regulation of the MAPK pathway can predispose cells to undergo tumorigenic transformation, as illustrated by the position of the *ras* oncogene upstream of ERK (Zhang *et al*, 1993). Transfection with constitutively active forms of MEK resulted in transformation, increased sensitivity to, or independence from growth factors *in vitro*, and tumorigenicity *in vivo* (Brunet *et al*, 1994; Mansour *et al*, 1994). A few studies have assessed the expression of activated MAPK in different clinical tumour specimens, and asked if this has any clinical or biological correlation. In renal cell carcinoma, MAPK activation correlated with MAPK kinase activation and Raf-1 activation (Oka *et al*, 1995), while for hepatocarcinomas a relationship was reported between ERK1/2 activation and expression of the transcription factor c-Fos and cyclin D1 (Ito *et al*, 1998). High levels of activated MAPK were found in high grade and advanced stage prostate cancers, suggesting a link between elevated ras signalling and advanced disease (Gioeli *et al*, 1999). Activated MAPK was also reported in glial tumours and in a group of oligodendrogliomas, an increase in the proportions of cells expressing activated MAPK corresponded with malignant progression (Mandell *et al*, 1998). Sivaraman *et al* (Itoh *et al*, 2002) provided the first demonstration of MAPK activation in human breast cancer tissues, comparing primary breast cancer with benign lesions using substrate-based MAPK enzyme assays and immunoblotting. They found significantly less MAPK activity to be in benign breast tissues compared with invasive breast cancers. In another study, a 2.5-fold increase in activated MAPK was seen in breast cancer specimens compared to normal breast tissue, and this was associated with poor prognosis and decreased sensitivity to endocrine therapy, and with expression of phosphorylated c-jun, a transcription factor activated by MAPK (Gee *et al*, 2000; Donovan *et al*, 2001). Phosphorylated MAPK is also thought to be involved in acquisition of resistance to antioestrogen treatment in ER-positive cells, as it has been shown to be one of the characteristics of advanced breast cancer and involved in the progression to oestrogen-independence (Jeng *et al*, 2000; Gee *et al*, 2001). In a recent study of human breast cancer specimens, activated MAPK detected by immunohistochemistry was increased in lymph node metastases compared with primary tumours, suggesting a role in the metastatic process (Adeyinka *et al*, 2002).

The causes of MAPK activation in human cancers vary among the different types of cancer. In many, ERK activation reflects the activity of mutated forms of *ras*. However, in breast cancer activating *ras* mutations are relatively rare, reported in only 5% of cases (Bos, 1989; Dickson *et al*, 1991), leading to the view that *ras* mutations do not have an important role in this disease. However, it is not easy to distinguish whether an apparent constitutive elevation in basal activity of ERK1/2 is due to an inherent alteration in the pathway regulation, or if the pathway is more sensitive to stimulation by an exogenous ligand. One possible explanation can still be connected to *ras*, as any one of the three major genes *(H-ras, K-ras, N-ras*) may be overexpressed in breast cancer, and this correlates with cancer progression (Von Lintig *et al*, 2000). Wild-type ras is subject to regulation by GTPase activating proteins and guanine nucleotide exchange factors by different upstream receptors, one of which is EGFR. A correlation between overexpression of EGFR and enhanced ERK activity has been previously suggested (Xing and Imagawa, 1998), and the present study explores the relationship further. The results, using several different cell lines in an attempt to mimic the heterogeneity of clinical breast cancer cases, do not fully support the previous report. We found that the levels of activated ERK1/2 did not uniformly correlate with EGFR or HER2 status in the different cell lines, and that different combinations of EGFR and low pERK1/2, or low EGFR and high pERK1/2 were found in the panel of cell lines studied. The results suggest that although EGFR and HER2 can signal through ERK1/2, an increase in receptors does not necessarily result in sustained ERK1/2 activity. Also, the hyperactivity of ERK1/2 in some of the cells studied may be secondary to other genetic, or epigenetic, alterations, for example the *K-ras* mutation in the MDA-MB-231 cell line (Davidson *et al*, 1987). Another potential explanation for elevation in ERK1/2 activation is a change in the expression or activation of specific threonine/tyrosine phosphatases that inactivate pERK1/2 (Tonks and Neel, 1996; Cook *et al*, 1997). Recently, it was shown that the expression of the MKP-1 phosphatase in Rat-1 cells was controlled by growth factors acting via ERK- and calcium-dependent pathways. Treatment with the phosphatase inhibitor sodium orthovanadate elevated basal ERK1/2 activity in the absence of growth factors (Cook *et al*, 1997). Thus, an attenuation of phosphatase expression or activity might be manifest as a rise in basal ERK1/2 activation.

Administration of PKI166 has been shown to be effective in controlling tumour growth and metastasis when used in combination with chemotherapy agents in preclinical models, including pancreatic cancer (Bruns *et al*, 2000), bladder and renal cell cancer (Baker *et al*, 2002). While used to target EGFR phosphorylation, PKI166 also inhibits phosphorylation of HER2 (Traxler *et al*, 2001). In this study the high HER2-expressing SKBR3 cell line was one of the more sensitive to growth inhibition and apoptosis induced by PKI166. As previously reported (Moasser *et al*, 2001; Bishop *et al*, 2002), and supported by the results of this study, a relatively high level of the target is needed to see the efficacy of an anti-EGFR agent. Similar to these previous reports, we also found that not every cell line expressing high levels of EGFR was growth inhibited by PKI166. Insensitive lines either had relatively little receptor expression or had a high basal activity of ERK1/2, the latter of which appeared to protect the cells from EGFR-signalling blockade. The prediction from this in the clinical situation is that while some tumours expressing high levels of EGFR will respond well to EGFR inhibitors, others may not. Determination of the levels of pERK1/2, in addition to expression of EGFR, in pretreatment biopsies may allow prediction of the response of a patient to anti-EGFR therapy.

Blocking EGFR signalling has been shown to stabilise the cyclin-dependent kinase inhibitor p27^KIP1^ and leads to G_1_ cell cycle arrest (Busse *et al*, 2000; Di Gennaro *et al*, 2003). PKI166 alone, and in combination with U0126 reduced ERK1/2 activity, and induced p27^KIP1^ expression in SUM149 cells. In contrast, only treatment with U0126 was able to induce G_1_ arrest and p27^KIP1^ in MDA-MB-231 cells, which were relatively insensitive to PKI166 alone. U0126 blocks MAPK signalling pathways by preventing activation of MEK1/2 (Davies *et al*, 2000), and it is also reported to block ERK5/BMK1 phosphorylation at the concentration used in this study (Kamakura *et al*, 1999). Signalling through ERK5 contributes to cyclin D1 regulation in breast cancer cells (Mulloy *et al*, 2003). Thus, the antiproliferative effects of U0126 may be due to the inhibition of ERK1/2 and ERK5 mediated signalling.

This study focused on the consequences of blocking EGFR and ERK signalling. However, even in the most sensitive cell lines, such as SUM149 and MDA-MB-468, in which ERK1/2 phosphorylation was almost completely inhibited, the growth inhibition or apoptosis induction were not more than 50–80% of control values. One explanation is that additional signalling pathways, insensitive to either PKI166 or U0126, were maintaining the viability of the cells. The phosphoinositide 3-kinase (PI3K) pathway is sensitive to EGFR-signalling blockade, promoting cell death through inhibition of AKT (Busse *et al*, 2000; Anderson *et al*, 2001; Moasser *et al*, 2001; Clark *et al*, 2002). PKI166 reduced basal levels of AKT activity in SUM 149, SKBR3 and MDA-MB-468, and inhibited EGF-induced AKT activity in MDA-MB-231 cells (data not shown). Similar to what this study found for ERK1/2 activity, the activation status of the PI3K/AKT pathway may influence responses to inhibitors. For example, Moasser *et al* (2001) found that MDA-MB-468 cells were relatively resistant to the tyrosine kinase inhibitor ZD1839, and this was attributed to the high basal AKT activity, resulting from deletion of the PTEN tumour suppressor (Lu *et al*, 1999).

The present study underscores the fact that the overexpression of EGFR or HER2 does not predict sensitivity to a therapy targeted to these receptors. Moreover, strategies designed to block more than one protein or pathway are likely to potentiate antiproliferative responses. Considering that cells in advanced breast cancers can have multiple mutations and genetic alterations, it is likely that therapeutic combinations targeting multiple pathways or key proteins will be more effective than single or nontarget-specific agents. Although ERK1/2 are not abnormal proteins, expression at abnormally high and sustained levels may be a potential target for pharmacological intervention for proliferative diseases, including cancer. The blockade of the MAPK pathway with an MEK inhibitor given orally suppressed the growth of colon tumours transplanted in mice, with no apparent side effects (Sebolt-Leopold *et al*, 1999). In our study, the combination of U0126 with PKI166 resulted in significant growth inhibition and apoptosis in cells expressing EGFR and pERK1/2. These results suggest that there is a strong molecular rationale supporting the continued development of inhibitors of the MAPK pathway, and for using them in combination with inhibitors of growth factor receptors such as the tyrosine kinase inhibitor PKI 166.
